# Supplementary material for: Lactobacillus paracasei Expressing Porcine Trefoil Factor 3 and Epidermal Growth Factor: A Novel Approach for Superior Mucosal Repair
Source: Vet Sci. 2025 Apr 14;12(4):365. doi: 10.3390/vetsci12040365 (PMC12031595; doi:10.3390/vetsci12040365)
Supplement: Supplementary file 1 [file vetsci-12-00365-s001.zip › vetsci-3519735-supplementary.pdf]

Supplementary Materials:

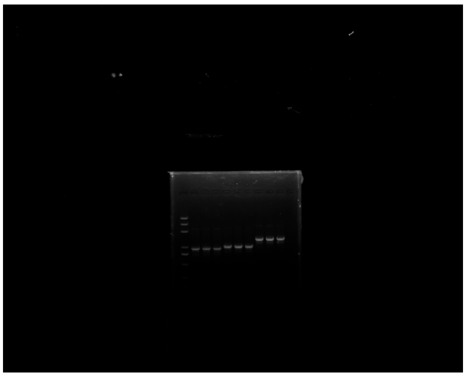

Figure S1. PCR identification results of recombinant *Lactobacillus.*

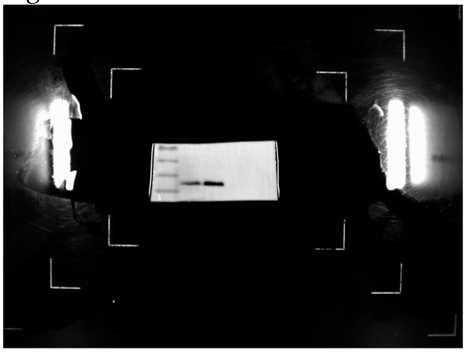

Figure S2. pTFF3-Western blot.

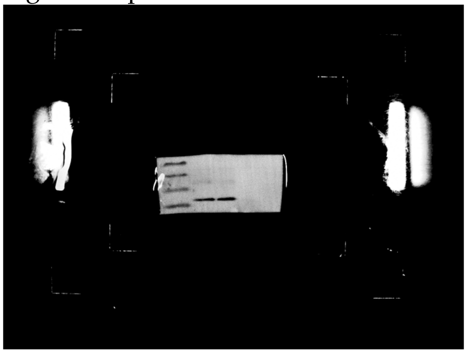

Figure S3. pEGF-Western blot.

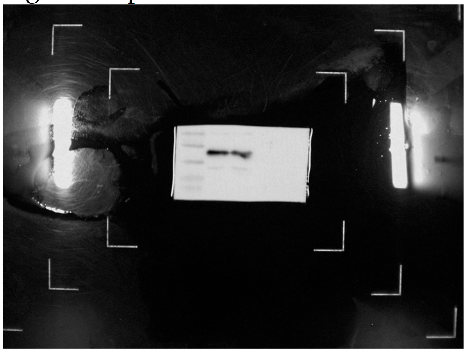

Figure S4. pTE-Western blot.

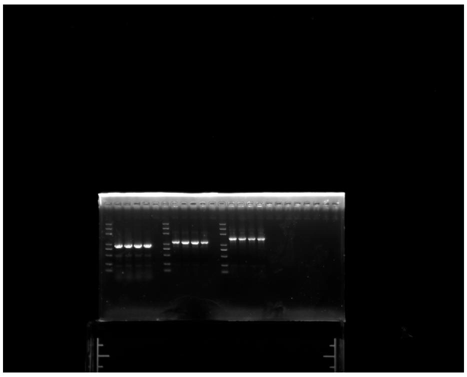

Figure S5. Stability analysis.

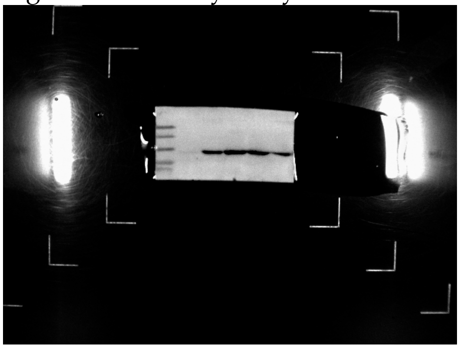

Figure S6. Stability analysis-pTFF3.

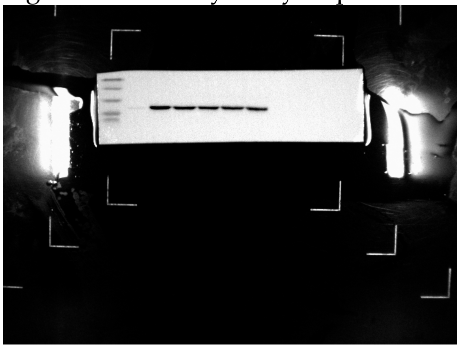

Figure S7. Stability analysis-pEGF.

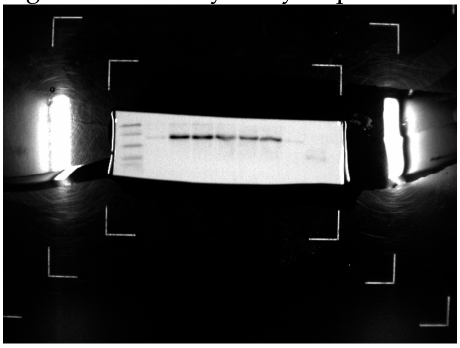

Figure S8. Stability analysis-pTE.

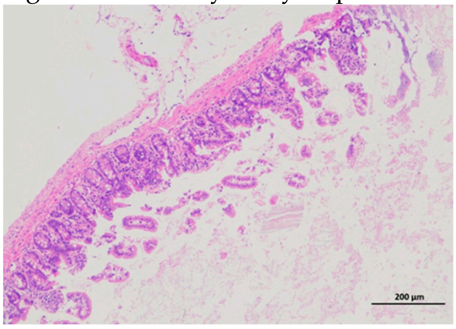

Figure S9. H&E-DSS.

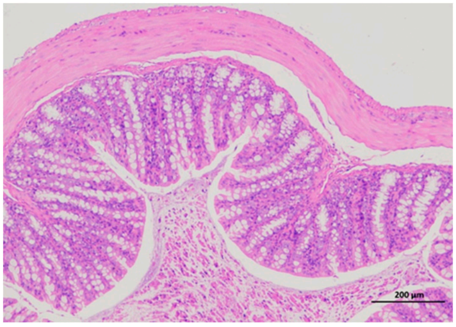

Figure S10. H&E-pPG/27-2.

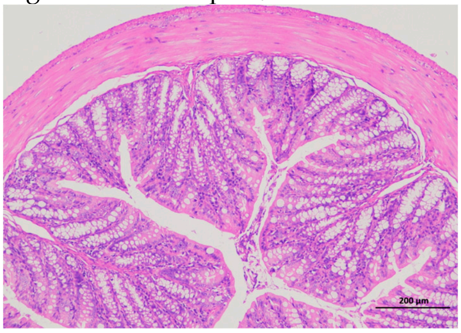

Figure S11. H&E-pPG-pTE/27-2.

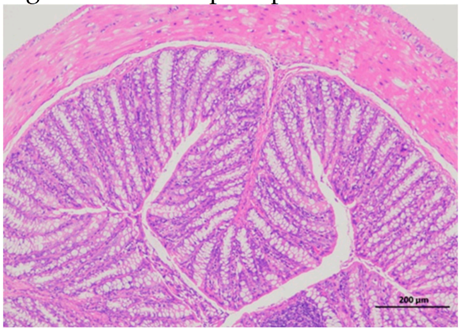

Figure S12. H&E-PBS.

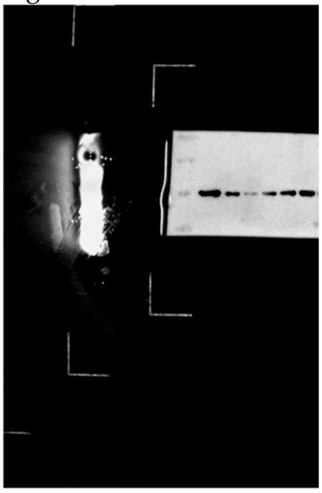

Figure S13. Western blot-Occludin.

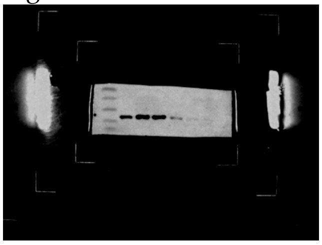

Figure S14. Western blot-Claudin-2.

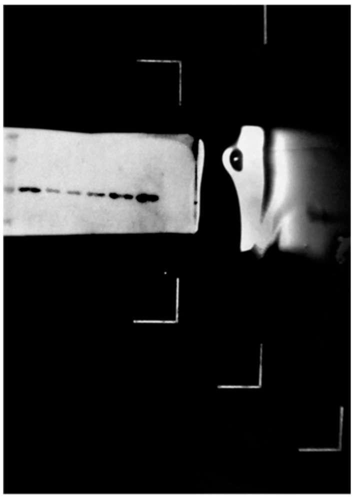

Figure S15. Western blot-ZO-1.

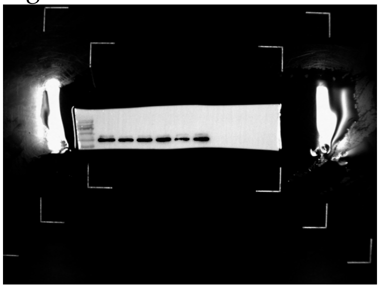

Figure S16. Western blot- β -actin.
